# Overexpression of matrix-metalloproteinase-9 in human breast cancer: a potential favourable indicator in node-negativeatients

**DOI:** 10.1054/bjoc.2001.1810

**Published:** 2001-06

**Authors:** A Scorilas, A Karameris, N Arnogiannaki, A Ardavanis, P Bassilopoulos, T Trangas, M Talieri

**Affiliations:** ‘G.Papanikolaou’, Research Center of Oncology; Department of Pathology; Department of Oncology; Department of Surgery, ‘St. Savas’ Hospital, 171 Alexandras Avenue, Athens 11522, Greece; Department of Pathology, 401 Army General Hospital, Athens, Greece; Present address: National Center of Scientific Research ‘Demokritos’, IPC, Athens 15310, Greece

**Keywords:** MMP-9, breast cancer, prognostic factor

## Abstract

Matrix metalloprotease-9 (MMP-9; 92 kDa type IV collaganase, gelatinase B) is regarded as, important for degradation of the basement membrane and extracellular matrix during cancer invasion and other tissue-remodelling events. In this study we evaluate the prognostic value of MMP-9, by immunoperoxidase staining in a series of 210 breast cancer tissues. The results were quantitated using the HSCORE system, which consider both staining intensity and the percentage of cells stained at given intensities. MMP-9 status was compared with the concentration of cytosolic Cathepsin-D and with other established prognostic factors, in terms of disease free survival and overall survival. The median follow-up period was 62 months. MMP-9 staining was observed primarily in cancer cells, and to a lesser degree in surrounding stromal cells. MMP-9 expression was not detected in normal breast tissue. Levels of MMP-9 expression below the cut-off point were more frequently observed in larger (*P* = 0.014), invasive ductal histologic (*P* = 0.037), progesterone receptor (PR)-negative and PR-strong positive tumours (*P*< 0.001), as well as samples belonging to patients with stage III-IV disease (*P* = 0.009) and age 45–55 years (*P* = 0.011). In univariate analysis, node-negative breast cancer patients with tumors positive for MMP-9 had a considerable reduction in risk for relapse (RR = 0.45;*P* = 0.039) or death (RR = 0.32;*P* = 0.009). Multivariate analysis indicated that MMP-9 status was an independent favourable predictor of OS (RR = 0.47;*P* = 0.034) in node-negative but not in node-positive patients. Our results suggest that MMP-9 may be an independent favourable prognostic factor in node-negative breast cancer patients. The overexpression of MMP-9 in breast cancer may be also used as a marker to subdivide node negative breast cancer patients in order to determine the optimal treatment modality. © 2001 Cancer Research Campaign http://www.bjcancer.com


*British Journal of Cancer* (2001) **84**(11), 1488–1496
© 2001 Cancer Research Campaign
doi: 10.1054/ bjoc.2001.1810, available online at http://www.idealibrary.com on IDEAL®

http://www.bjcancer.com

### Overexpression of matrix-metalloproteinase-9 in human breast cancer: a potential favourable indicator in node-negative patients

**A Scorilas[1,5,6], A Karameris[5], N Arnogiannaki[2], A Ardavanis[3], P Bassilopoulos[4], T Trangas[1] and M Talieri[1]**

[1]'G.Papanikolaou' Research Center of Oncology; [2]Department of Pathology; [3]Department of Oncology; [4]Department of Surgery, 'St. Savas' Hospital, 171 Alexandras Avenue, Athens 11522, Greece; [5]Department of Pathology, 401 Army General Hospital, Athens, Greece; [6]Present address: National Center of Scientific Research 'Demokritos', IPC, Athens 15310, Greece

**Summary** Matrix metalloprotease-9 (MMP-9; 92 kDa type IV collaganase, gelatinase B) is regarded as, important for degradation of the basement membrane and extracellular matrix during cancer invasion and other tissue-remodelling events. In this study we evaluate the prognostic value of MMP-9, by immunoperoxidase staining in a series of 210 breast cancer tissues. The results were quantitated using the HSCORE system, which consider both staining intensity and the percentage of cells stained at given intensities. MMP-9 status was compared with the concentration of cytosolic Cathepsin-D and with other established prognostic factors, in terms of disease free survival and overall survival. The median follow-up period was 62 months. MMP-9 staining was observed primarily in cancer cells, and to a lesser degree in surrounding stromal cells. MMP-9 expression was not detected in normal breast tissue. Levels of MMP-9 expression below the cut-off point were more frequently observed in larger ($P = 0.014$), invasive ductal histologic ($P = 0.037$), progesterone receptor (PR)-negative and PR-strong positive tumours ($P < 0.001$), as well as samples belonging to patients with stage III-IV disease ($P = 0.009$) and age 45–55 years ($P = 0.011$). In univariate analysis, node-negative breast cancer patients with tumors positive for MMP-9 had a considerable reduction in risk for relapse (RR = 0.45; $P = 0.039$) or death (RR = 0.32; $P = 0.009$). Multivariate analysis indicated that MMP-9 status was an independent favourable predictor of OS (RR = 0.47; $P = 0.034$) in node-negative but not in node-positive patients. Our results suggest that MMP-9 may be an independent favourable prognostic factor in node-negative breast cancer patients. The overexpression of MMP-9 in breast cancer may be also used as a marker to subdivide node negative breast cancer patients in order to determine the optimal treatment modality. © 2001 Cancer Research Campaign http://www.bjcancer.com

**Keywords**: MMP-9; breast cancer; prognostic factor

The aggressiveness of a tumour is primarily dependent on its ability to invade adjacent tissue and to metastasize to distant sites. Proteolytic degradation of the basement membrane and the extracellular matrix is a central aspect of physiologic and pathologic tissue-remodelling processes, such as neovascularization, wound healing, and malignant growth. Various proteolytic enzymes such as matrix-metalloproteinases (MMPs), serine proteinases and cathepsins have been implicated in the proteolytic process (Dano et al, 1994; Chambers et al, 1997; Benaud et al, 1998; Yan et al, 1998; Nelson et al, 2000; Scorilas et al, 2000a and b; Magklara et al, 2000; Yousef et al, 2000; Yousef et al, 2001). Digestion of the subendothelial basement membrane is the first step towards intravasation and extravasation of cancer cells. Type IV collagen is the main component of the basement membrane and degradation of this structural protein is favoured by two metalloproteases, namely the 72kDa type IV collagenase (MMP-2, gelatinase A) and the 92kDa type IV collagenase (MMP-9, gelatinase B).

The in vivo origin of MMPs remains ambiguous, since MMP-2 production has been observed in fibroblasts, osteoblasts, endothelial cells and macrophages, while MMP-9 has been observed in polymorphonuclear leukocytes, vascular pericytes, macrophages and keratinocytes (Nielsen et al, 1996a, 1997; Sugiura et al, 1998). In breast cancer, immunohistochemical studies have indicated that MMP-2 is expressed in both normal and malignant breast tissue and MMP-9 in macrophages located adjacent to the invading tumour front (Tryggvason et al, 1993) and tumour cells (Jones et al, 1999). MMPs are secreted as inactive proenzymes. Pro-MMP-2 is activated in vitro on the cell surface by a membrane-type matrix metalloprotease (MT-MMP) (Strongin et al, 1995). Several enzymes can activate pro-MMP-9 in vitro, including interstitial collagenase (MMP-1), stromelysin-l (MMP-3), matrilysin (MMP-7) and MMP-2 (Friedman et al, 1995); nevertheless, little is known about the activation mechanism in vivo. Activity of MMPs is also regulated by tissue inhibitors of metalloproteases (TIMPs) (Blavier et al, 1996). In vivo expression studies of the MMPs and TIMPs indicate that stromal cells in several types of cancer contribute by synthesizing these proteins (Hewitt et al, 1996; Heppner et al, 1996; Sugiura et al, 1998). Furthermore, components of the plasminogen activation system, such as PAI-1, are expressed by stromal cells in breast cancer (Bianchi et al, 1995; Nielsen et al, 1996b). The imbalance between MMPs and TIMPs resulting from increased production and activation of MMPs plays an important role in the invasiveness of the cancer (Liotta et al, 1991).

Several MMP family members have been specifically implicated in the progression of breast cancer. However, the regulation

*Received 15 June 2000*
*Revised 22 February 2001*
*Accepted 28 February 2001*

*Correspondence to:* M Talieri of MMP expression and activity in tumours appears complex and the manner in which MMPs collectively contribute to breast tumour progression is not well understood. In general, the induction of MMPs in the stroma within tumours and in adjacent normal tissue represents a direct or indirect host response to the presence of tumour cells (Heppner et al, 1996). With regard to MMP-9, many reports show that it is expressed in breast cancer tissue, but there are conflicting results regarding the identity of the cells that express this protease (Visscher et al, 1994; Heppner et al, 1996; Nielsen et al, 1996a, 1997). Although the expression of MMP-9 in breast tumour tissue has been examined by different methods, namely ELISA (Duffy et al, 1995), northern blotting (Pacheco et al, 1998), zymography (Rha et al, 1997, 1998), in situ hybridization (Nielsen et al, 1997) and immunohistochemistry (Nielsen et al, 1997) but to the best of our knowledge, survival analysis connecting MMP-9 expression with disease outcome has not been performed with enough samples to produce statistically significant results.

In this study we examined the expression of MMP-9 using immunocytochemistry in 210 Greek cancer patients, correlated it with clinicopathological disease variables, and evaluated its prognostic value. To our surprise, this expression was associated with pathologic and biochemical features of less aggressive disease, and with a favourable prognosis. Similar reports by other groups have attributed favorable prognosis to the expression of other proteases like PSA (Yu et al, 1998) and Pepsinogen C (Scorilas et al, 1999c) in breast cancer patients, although their findings have not been fully explained until now.

### MATERIALS AND METHODS

### Tissue samples and patients

Tumour samples from 210 patients who underwent surgery for primary breast cancer between 1989 and 1993 at the Oncologic Hospital of Athens 'St Savas' and at the '401 Army Hospital' were evaluated in this study. Ten breast tissue samples from individuals confirmed to be free of malignancy were analysed for negative control purposes. Tumour specimens were randomly drawn from a pool of frozen specimens originally submitted to the Laboratory of Hormone Receptors for steroid receptor analysis. A computerized database of updated patient clinical data, in addition to receptor status, nodal status and number of positive nodes, primary tumour diameter, age and menopausal status of the patients, and/or differentiation grade of the tumour, was available for statistical analysis.

All patients had a histologically confirmed diagnosis of primary breast cancer and received no treatment before surgery. Surgery consisted of modified mastectomy (87 patients) or breast conserving lumpectomy (123 patients). Axillary lymph node dissection was performed on 96% of the patients. For these patients, the positivity rate for cancer involvement of lymph nodes was 57.7%. Tumour size ranged from 0.6 to 7.0 cm in diameter, the mean and median being 2.9 cm and 2.7 cm, respectively. Clinical staging was performed according to the Postsurgical International Union Against Cancer Tumour-Node-Metastasis classification system (Spiessl et al, 1989). Of the 206 patients for whom the stage was known, 24(11.6%), 135(65.5%), 42(20.4%) and 5(2.5%) were classified as Stage I, II, III and IV, respectively. Histologic grade of the tumours was known for 202 patients and was as follows, according to the criteria reported by Scarff–Bloom–Richardson (Doussal et al, 1989): 9 patients (4.5%) had grade I, 164 (81.2%) had grade II and 29 patients (14.3%) had grade III tumours. Most of the tumours for which the histologic type was known (178 patients) were invasive ductal (88.8%), whereas the remaining were invasive lobular (8.4%) and other histologic types (2.8%). The post-operative treatment modality was known for all patients; 4.8% received no further treatment after tumour resection, 28.0% were given adjuvant chemotherapy, 59.0% were treated with endocrine therapy, and 8.2% were given both chemotherapy and endocrine therapy. Postoperative locoregional radiotherapy was administered to 148 patients. Disease relapse was defined as the first documented evidence of local or regional axillary recurrence or distant metastasis.

Patient age ranged from 24 to 94 years; the median age being 56 years. Twenty-one percent of the patients were under the age of 45, 19% were between 45 and 55, and 60% were 56 years or older. Follow-up information was available for 206 patients and included survival and relapse status, with the dates of the events and cause of death, if applicable. The distribution of follow-up times for patients still alive at the time of analysis ranged from 24 to 83 months, with a median of 68 months; only 6 and 2 patients had been followed less than 48 and 36 months, respectively. Follow-up times for the entire cohort, however, ranged from 10 to 85 months and had a median of 62 months. The Relapse-free survival (RFS) time in each case was the time interval between the date of surgical removal of the primary cancer and the date of the first documented evidence of relapse. The overall survival (OS) time was the time interval between the date of surgery and the date of death, or the date of last follow-up for those who were alive at the end of the study.

### Immunohistochemistry

The indirect immunoperoxidase method was adopted for immunostaining using routinely processed paraffin embedded specimens. Tissue sections (5 μm in thickness) were microwave-treated for 10 min in PBS after deparaffinization and rehydration. Endogenous peroxidase activity was blocked with 3% $H_2O_2$ in methanol for 8 min, followed by two 5 min washes with PBS. The tissue sections were incubated with normal goat serum for 20 min, and then with the primary sheep anti-MMP-9 antibody (Biodesign International Cat # k90163C USA) at a working dilution of 1:1000. Fifty of the sections were stained as well with the polyclonal antibody MMP-9(C-20):sc6840(Santa Cruz Biotechnology). Sections were washed twice for 5 min with PBS and incubated for 30 min with a biotinylated secondary antibody (Vectastain ABC Elite Kit, Vector Laboratories, CA, USA) diluted 1:200 in PBS. Sections were washed twice for 5 min with PBS, developed for 5 min with 0.05% 3,3-diaminobenzidine tetrahydrochloride (DAB) (Sigma, UK), slightly counterstained with haematoxylin, dehydrated, cleared and mounted with DePex (BDH, Limited Poole, UK). Specificity of the immunocytochemical reaction was tested by pre-absorption of the anti-MMP-9 antibody with the purified antigen from polymorphonuclear leukocytes (Elashry–Stowers et al, 1988) which completely abolished the specific staining. Negative controls had the primary antibody omitted and replaced by PBS. MMP-9 immunoreactivity was evaluated using the Image Analysis System 2000 (Digital Image System Hellas) as described (Karameris et al, 1997). In each case, 20 optical fields of high magnification (× 400) were measured and 300 cells were counted. All samples were evaluated for MMP-9 expression by two pathologists who were unaware of patient

© 2001 Cancer Research Campaign outcome. Staining intensity was classified from 0 (no staining) to 3 (very strong staining). For each slide, a value designated HSCORE was obtained by application of the following algorithm: HSCORE $=\sum[(I + 1) \times PC]$, where I and PC represent intensity and percentage cells that stain at each intensity, respectively. Reproducibility of the scoring method between both observers was greater than 95%. In the remaining cases in which discrepancies had been noted, differences were established by agreement review of corresponding slides (Karameris et al, 1997; Vizoso et al, 1995).

### Immunoabsorption of MMP-9

Described previously by Elashry–Stowers et al, 1988, in brief, protein A-Sepharose (Sigma) was precoated with secondary rabbit-anti-mouse IgG (Dako) and unbound IgG was removed by washing. IgG-coated protein A-Sepharose was then incubated with MMP-9 antibody and then washed to remove any free antibody. Purified MMP-9 antigen(MMP-9, human neutrophil granuloside, Oncogene Science) was then incubated with antibody-coated protein A-Sepharose. After incubation protein A-Sepharose beads were washed. Beads were centrifuged and the supernatant used for immunohistochemical staining after concentration.

### Hormone receptors

Estrogen receptors (ER) and progesterone receptors (PR) were assayed by the charcoal method as previously described (Kute et al, 1980). Results were expressed as specific binding sites per mg of cytosolic protein (fmol/mg protein). Tumours with estrogen and progesterone receptor concentrations below or equal to 10 fmol/mg protein were considered receptor negative, tumours with receptor concentrations between 10 and 300 fmol/mg protein were considered positive, whereas concentrations above that value were characterized as strongly positive.

### Cathepsin-D

Cathepsin-D was assayed using an immunoradiometric kit (Elsa Cath-D kit. CIS Bio International. Gif-sur-Yvette, France) according to the procedure described by the manufacturer. Reconstituted tumour cytosols were analyzed in duplicate at dilutions of 1:40 and 1:80 as we described previously (Scorilas et al, 1993, 1995).

### Statistical analysis

An optimal cut-off point equal to 48th percentile was defined using chi-square ($\chi^2$) analysis based on the ability of MMP-9 expression to predict the RFS and OS for the population studied. Relationships between MMP-9 status and other variables were analyzed using the $\chi^2$ test, as well as the Fisher's exact test, where appropriate. ER and PR values were categorized into negative, positive and strongly positive as described above. Tumour size was classified as smaller than 2 cm in diameter, between 2 and 5 cm, or larger than 5 cm. Lymph node status was either positive (histological evidence of tumour extension to one or more lymph nodes) or negative. Patient age was categorized into three groups: less than 45 years, 45 to 55 years and greater than 55 years. Survival analyses were performed by constructing Kaplan–Meier RFS and OS curves (Kaplan and Meier, 1958), where differences between curves were evaluated by the log-rank test. Cox regression analyses using the SAS statistical software (SAS Institute, Cary, NC) was used to calculate the relative risk (RR) and 95% confidence interval (CI). Only patients for whom the status of all variables was known were included in the multivariate models, which incorporated MMP-9 status and all other variables for which the patients were characterized.

### RESULTS

### Expression of MMP-9 in breast tissues

MMP-9 expression in breast tissues was analysed by immunohistochemical staining using a sheep polyclonal antibody. In breast tumours, MMP-9 expression was observed primarily (~80%) in cancer cells, and secondly in stromal cells (Figures 1A and 1B). Morphological characterization suggested that three different stromal cell types showed MMP-9 positivity: neutrophils and macrophage-like cells in all samples, and vascular cells in 68% of samples. The expression of MMP-9 was homogeneous, cytoplasmically diffuse, and occasionaly membranous as seen by intense staining in parts of the membrane (Figure 1C).

### MMP-9 expression and relationship to other prognostic variables

MMP-9 status was evaluated in 210 breast tumours and 10 non malignant breast tissue samples. An optimal cut-off value was

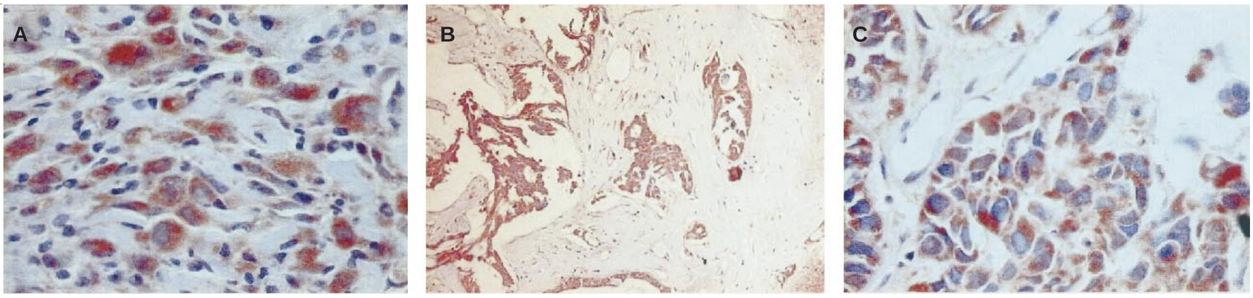

**Figure 1** (**A**) Cytoplasmic expression of MMP-9 by tumour cells in infiltrating ductal breast carcinoma. Immunohistochemical staining × 200, DAB staining. (**B**) Positive mainly tumour and few stromal cells against MMP-9, in infiltrating breast carcinoma. Immunohistochemical staining × 100, DAB chromogen. (**C**) Dense membranous and cytoplasmic staining against MMP-9 in tumour cells of breast carcinoma. Immunohistochemical staining × 250, DAB chromogen

 *© 2001 Cancer Research Campaign*

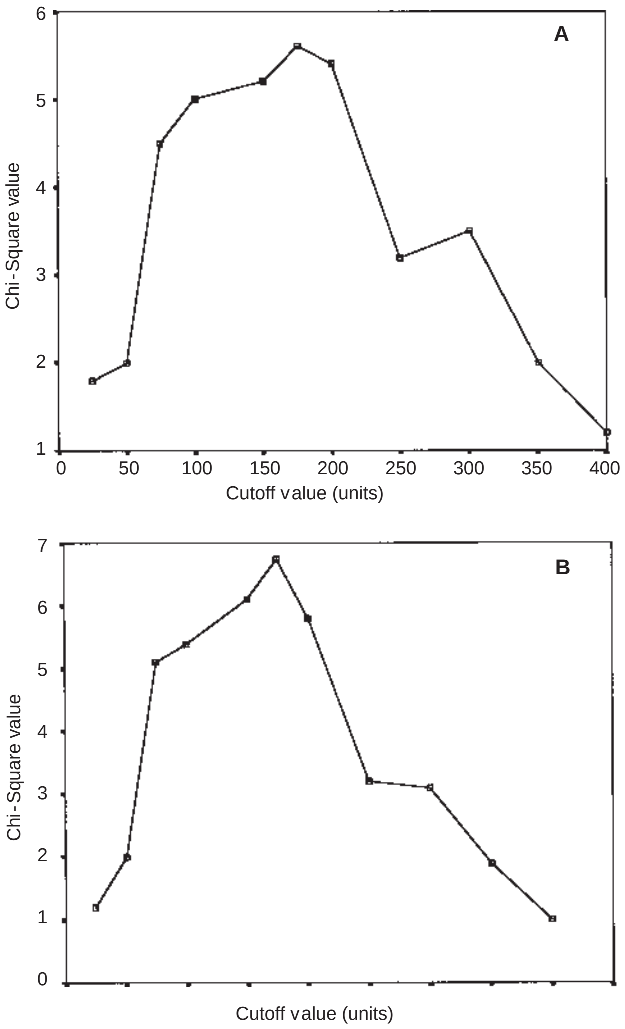

**Figure 2** Determination of optimal cut-off value of MMP-9 status for prediction of relapse-free (**A**) and overall survival (**B**) of breast cancer patients. The $\chi^2$ values obtained at each cut-off-value are plotted against the value itself. A HSCORE value of 175 is shown to be the optimal cutoff ($\chi^2$ = 5.61 and $\chi^2$ = 6.74 RFS and OS, respectively). This cut-off (48th percentile) identifies 52% of patients as being MMP-9 positive defined by $\chi^2$ analysis, based on the ability of MMP-9 expression to predict the RFS and OS for the population studied. As shown in Figure 2, HSCORE value of 175 is shown to be the optimal cut-off ($\chi^2$ = 5.61, $P$ = 0.02 and $\chi^2$ = 6.74, $P$ < 0.01 for RFS and OS, respectively). This cut-off (48th percentile) identifies 52% and 48% of tumours as being MMP-9 positive and MMP-9 negative, respectively. Over-expression of MMP-9 in normal breast tissue was not observed. MMP-9 negative tumours tended to be larger in size ($P$ = 0.014), histologically classified as invasive ductal ($P$ = 0.037), PR-negative and PR-strong positive tumours ($P$ < 0.001), and obtained from patients who were diagnosed with stage III–IV disease ($P$ = 0.009) and were between the ages of 45 and 55 ($P$ = 0.011). No significant association between MMP-9 status and tumour grade, nodal status, ER, and Cathepsin D was observed (Table 1).

### MMP-9 status and breast cancer survival

Univariate and Multivariate Analysis. Follow-up information was available for 206 of the 210 patients included in the study. During their respective follow-up periods, 77 patients (37.3%) relapsed and 49 (23.8%) died. Cox univariate survival analysis revealed that, the risk for relapse and death was significantly weak in relation to MMP-9 considered as a dichotomous variable (Table 2). These regression models showed that there was a reduction in risk for relapse and death in patients whose tumours showed MMP-9 positivity, compared to those which were MMP-9 negative. The Kaplan-Meier survival curves (Figure 3) also showed that MMP-9 positivity was associated with relapse-free survival(RFS) and overall survival(OS). In multivariate analysis, the Cox regression models were adjusted for age, nodal status, tumor size, tumor grade, Cathepsin-D, ER, and PR, all of which were used as categorical variables, except tumour size, which was used as a continuous variable, as described above. Patient age, tumour size, ER status and nodal status were thus shown to be independent factors for predicting both RFS and OS. MMP-9 did not add significantly to the prognostic power in the multivariate model for RFS and OS.

### Univariate and multivariate analysis for patients classified by nodal status

Since node-positive patients are substantially different from those which are node-negative in terms of their prognosis and treatment administered after surgery, univariate and multivariate Cox regression models were used to evaluate the effect of MMP-9 on RFS and OS for each of the two groups of patients. The results are shown in Table 2 and Figure 4. Node-negative breast cancer patients with tumours positive for MMP-9 tended to have a considerable reduction in risk for relapse (RR = 0.45; $P$ = 0.039) or death (RR = 0.32; $P$ = 0.009). According to multivariate analysis: patient age, tumour size and Cathepsin-D were found to be independent factors for predicting both RFS and OS. MMP-9 significantly added to prognostic power in the multivariate model of analysis for OS (RR = 0.47; $P$ = 0.034). When the relationship between MMP-9 and survival was examined in node-positive patients, none of the differences were statistically significant.

### DISCUSSION

To the best of our knowledge, this is the first paper to report an inverse association between MMP-9 overexpression in breast cancer and favourable prognosis in node negative patients. Using immunohistochemistry, we have revealed that node negative breast cancer patients whose tumours overexpress MMP-9 have a considerable reduction in the risk for relapse or death. These unexpected results are difficult to explain in terms of the current concept of the molecular mechanisms involved in tumour invasion and metastasis. Another paradox was the poor prognosis associated with high levels of PAI-1 in breast cancer, recently resolved by Bajou et al (1998) demonstrating that host-produced PAI-1 is essential for cancer cell invasion and angiogenesis. The authors (Ree et al, 1998) tried to explain the apparent paradox by accepting that PAI-1 serves as a tumour promoter, e.g. by protecting the tumour stroma, to which PAI-1 has been localized in breast cancer lesions (Bianchi et al, 1995), from ongoing uPA-mediated proteolysis.

As shown recently (Takeha et al, 1997) in colon cancer, host inflammatory cells expressing MMP-9 and uPAR were less prevalent in cases with liver metastasis than in cases without liver metastasis. Furthermore, expression was lower in cases with an infiltrating margin than in cases with an expanding margin. These

**Table 1** Relationship between MMP-9 status and other variables

| | | No. of patients (%) | | |
|---|---|---|---|---|
| **Variable** | **Patients** | **MMP-9 negative** | **MMP-9 positive** | ***P* value** |
| Age (years) | | | | |
| <45 | 44 | 13 (29.5) | 31 (70.5) | |
| 45–55 | 39 | 24 (61.5) | 15 (38.5) | 0.011[e] |
| >55 | 127 | 63 (49.6) | 64 (50.4) | |
| Tumour size (cm) | | | | |
| ≤2 | 48 | 23 (47.9) | 25 (52.1) | |
| 2–5 | 138 | 59 (42.8) | 79 (57.2) | 0.014[e] |
| >5 | 24 | 18 (75.0) | 6 (25.0) | |
| Nodal status | | | | |
| Negative | 85 | 43 (50.6) | 42 (49.4) | 0.34[f] |
| Positive | 116 | 54 (46.6) | 62 (53.4) | |
| x | 9 | | | |
| Grade[a] | | | | |
| I | 9 | 4 (44.4) | 5 (55.6) | |
| II | 164 | 83 (50.6) | 81 (49.4) | 0.27[e] |
| III | 29 | 10 (34.5) | 19 (65.5) | |
| × | 8 | | | |
| Histology | | | | |
| Ductal | 158 | 81 (51.3) | 77 (48.7) | |
| Lobular | 15 | 5 (33.3) | 10 (66.7) | 0.037[e] |
| Other | 5 | 0 (0.0) | 5 (100.0) | |
| × | 32 | | | |
| Stage[b] | | | | |
| I | 24 | 10 (41.7) | 14 (58.3) | |
| II | 135 | 58 (43.0) | 77 (57.0) | 0.009[e] |
| III–IV | 47 | 32 (68.1) | 15 (31.9) | |
| × | 4 | | | |
| ER status[c] | | | | |
| Negative | 35 | 14 (40.0) | 21 (60.0) | |
| Positive | 151 | 70 (46.4) | 81 (53.6) | 0.19[e] |
| Strong positive | 20 | 13 (65.0) | 7 (35.0) | |
| × | 4 | | | |
| PR status[c] | | | | |
| Negative | 28 | 20 (71.4) | 8 (28.6) | |
| Positive | 138 | 50 (36.2) | 88 (63.8) | <0.001[e] |
| Strong positive | 40 | 27 (67.5) | 13 (32.5) | |
| × | 4 | | | |
| Cathepsin D status[d] | | | | |
| Negative | 104 | 53 (50.1) | 51 (49.9) | 0.21[f] |
| Positive | 106 | 47 (44.3) | 59 (55.7) | |
| Adjuvant treatment | | | | |
| None | 10 | 6 (60.0) | 4 (40.0) | |
| Tamoxifen | 124 | 66 (53.2) | 58 (46.8) | 0.057[e] |
| Chemotherapy ± tamoxifen | 76 | 28 (36.8) | 48 (63.2) | |

[a]Bloom–Richardson grading system; [b] TNM system; [c]Negative: < 10 fmol/mg; Positive: 10–300 fmol/mg; Strong positive: > 300 fmol/mg. [d] Cut-off point: 60 pmol/mg; [e] $\chi^2$ test; [f]Fisher's Exact Test.

results indicate the presence of an inverse association between matrix degrading proteinases and liver metastasis or infiltrating growth pattern. The authors explain their data by proposing that the immune/inflammatory reaction, forming a part of the host's defence mechanisms, is a significant source of matrix-degrading proteinases. According to their report, MMP-9 was immunohistochemically positive in macrophages and neutrophils, but negative in cancer cells. The authors, however, do not exclude the possibility that cancer cells are one of the major sources of MMP-9 protein, since results may vary depending on the epitopes of each antibody used. In our study, MMP-9 staining was mainly observed in cancer cells (~80%) and less in stromal fibroblasts, macrophages and neutrophils, whereas Nielsen et al (1997) showed that detectable amount of MMP-9 protein is absent from cancer cells in ductal mammary carcinoma and is expressed only in neutrophils, macrophages and vascular pericytes. Our findings agree with the results of Visscher et al (1994) who found that 64% of tumours showed staining of epithelial cells with the MMP-9 Mab, although he observed 20% of staining in patients with node negative disease in contrast to our 49.4%. However, several MMPs including gelatinase B / MMP-9 (Vu and Werb, 1998) are secreted and mechanisms that enable their association with the cell membrane are largely unknown.

Recently Yu and Stamenkovic (1999) showed that CD44 serves as an MMP-9 docking molecule to retain MMP-9 proteolytic activity on the cell surface, providing evidence that CD44/MMP-9

 *© 2001 Cancer Research Campaign*

**Table 2** Association between MMP-9 expression and relapse-free and overall survival

| | Relapse-free survival | | | Overall survival | | |
|---|---|---|---|---|---|---|
| **MMP-9 status** | **RRa** | **95% CIb** | **P value** | **RRa** | **95% CIb** | **P value** |
| *Univariate analysis* | | | | | | |
| All patients (*n* = 206) | | | | | | |
| MMP-9 negative | 1.00 | | | 1.00 | | |
| MMP-9 positive | 0.65 | 0.41–1.04 | 0.072 | 0.59 | 0.33–1.06 | 0.078 |
| Node-positive patients (*n* = 116) | | | | | | |
| MMP-9 negative | 1.00 | | | 1.00 | | |
| MMP-9 positive | 0.89 | 0.47–1.67 | 0.73 | 1.14 | 0.49–2.96 | 0.63 |
| Node-negative patients (*n* = 85) | | | | | | |
| MMP-9 negative | 1.00 | | | 1.00 | | |
| MMP-9 positive | 0.45 | 0.21–0.96 | 0.039 | 0.32 | 0.14–0.75 | 0.009 |
| *Multivariate analysis* [c] | | | | | | |
| All patients (*n* = 202) | | | | | | |
| MMP-9 negative | 1.00 | | | 1.00 | | |
| MMP-9 positive | 0.89 | 0.49–1.61 | 0.71 | 0.78 | 0.35–1.75 | 0.55 |
| Node-positive patients (*n* = 113) | | | | | | |
| MMP-9 negative | 1.00 | | | 1.00 | | |
| MMP-9 positive | 0.99 | 0.45–2.18 | 0.99 | 0.97 | 0.33–2.85 | 0.96 |
| Node-negative patients[d] (*n* = 84) | | | | | | |
| MMP-9 negative | 1.00 | | | 1.00 | | |
| MMP-9 positive | 0.23 | 0.04–1.31 | 0.098 | 0.47 | 0.24–0.94 | 0.034 |

[a]Relative risk (RR) estimated from Cox proportional hazard regression model. [b]Confidence interval of the estimated RR.
[c]Multivariate models were adjusted for lymph nodes status; tumour size; patient age; cathepsin D; ER and PR expression.
[d]Including also in the multivariate model the stage of the disease.

complex formation is associated with tumor invasiveness in vivo. Also, Sugiura et al (1998) upon examining the expression of MMP-9 in human neuroblastoma revealed the presence of MMP-9 in stromal septa that separated nests of tumour cells. These septa consisted of fibroblasts, vascular and perivascular cells, but not of neuroblastic cells. They concluded that the matrix-degrading proteolytic activity of a tumour is the result of a complex balance between several factors including proMMP-9, active MMP-9, MMPactivators, TIMPs and the balance is itself controlled by interactions between tumour cells and host-derived stromal cells. Recently, Mira et al (1999) showed that MMP-9 is expressed by MCF-7 cells and is located on the cell surface as well as in the conditioned medium. They hypothesized that IGF-1 promotes a redistribution of an MMP-9 pool between the cell surface and the medium and highlighted the importance of cell surface-anchored MMP-9 activity in mediating IGF-1 chemoatractant activity. Also, Remacle et al (1998), using gelatin zymography, found no significant relationship between levels of either MMP-9 and breast patient outcome, while a 50 kDa band in their zymogram was associated with improved overall survival. Similarly, Visscher et al (1994) found no relationship between levels of either MMP-2 or MMP-9 and prognosis in breast cancer, while Daidone et al (1991) found no association between collagenase IV levels and either relapse-free survival or overall survival in axillary node-negative breast cancer patients. Nevertheless, Pacheco et al (1998) reported that overexpression of gelatinase B has a prognostic value for breast cancer patients.

In the present study, MMP-9-negativity was found more frequently in the age cohort of 45 to 55 years which is the age with the greater relative risk for relapse and death in relation to the ages < 45 years (RR = 1.6) and > 55 years (RR = 3.2) (data not shown). The expression of MMP-9 was positively associated in patients with positive progesterone receptors (10 –300 fmol/mg protein), but its expression was low in patients with strongly positive estrogen or progesterone receptors (> 300 fmol/mg protein). With regard to the latter observation, we have shown in the past that a high concentration of estrogen or progesterone receptors indicates poor prognosis in breast cancer (Ardavanis et al, 1997; Scorilas et al, 1999a, b). It has also been suggested by Thorpe et al (1993) that tumours with extremely high levels of ER may indicate a progression from a fully hormone-dependent to a hormone-independent state, via hormone responsive phase. Moreover, in vitro studies in other organs, such as the endometrium, have suggested that progesterone can suppress the expression of MMPs, although estradiol does not appear to directly activate their expression (Bruner et al, 1995). In the recent study by Salamonsen et al (1997) progesterone withdrawal from the endometrium has the potential both to increase the level of pro-MMP-1 within the tissue and to change the balance between the MMPs and tissue inhibitors of metalloproteinases that would be required for active tissue degradation.

We demonstrated in previous studies that the protease Cathepsin-D is a marker of poor prognosis for node negative breast cancer patients (Ardavanis et al, 1997; Scorilas et al, 1999a). In this study significant relationship between Cathepsin-D and MMP-9 was not observed. The present study suggests that the protease MMP-9 is a favourable prognosticator. In addition, the proteases PSA (Yu et al, 1998) and Pepsinogen-C (Scorilas et al, 1999c) have also been characterized as favourable prognosticators but for node positive breast cancer patients. In light of the fact that not all hormone receptors are functional, the authors (Yu et al, 1998; Scorilas et al, 1999c) proposed that both proteases may serve as better indicators of a functional pathway than the presence of the steroid hormone receptors themselves. Because 30% of node-negative breast cancer patients relapse, determination of MMP-9 expression may help to separate patients with better

© 2001 Cancer Research Campaign 

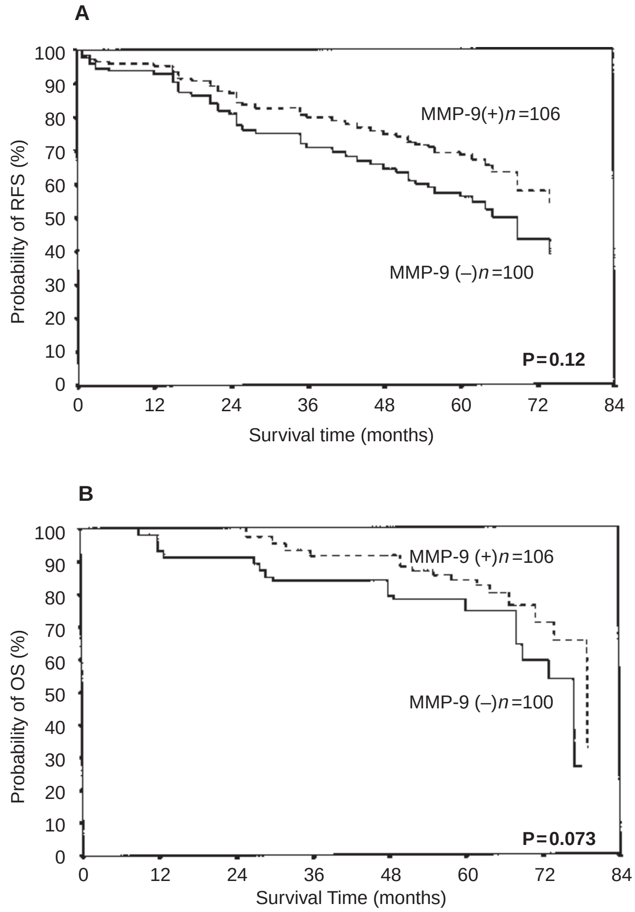

**Figure 3** Relapse-free (**A**) and overall survival (**B**) curves for patients with MMP-9 positive and MMP-9 negative breast tumour tissues, followed for a median of 62 months

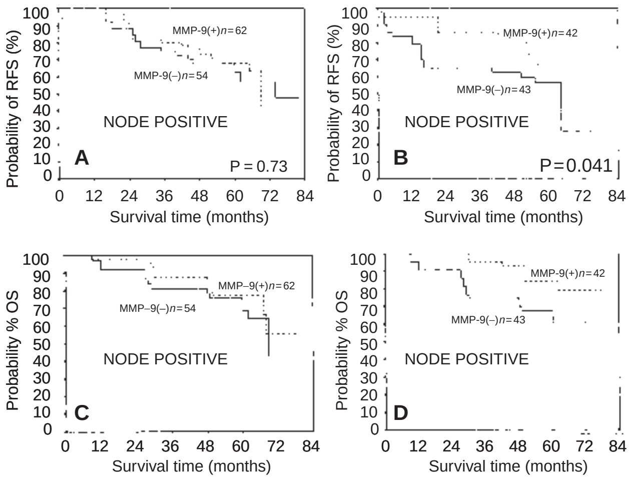

**Figure 4** Relapse-free (**A** and **B**) and overall survival (**C** and **D**) curves of patients with MMP-9 positive and MMP-9 negative breast cancer, stratified by their nodal status: node-positive (**A** and **C**); node-negative (**B** and **D**)

prognosis and allow the use of different treatment modalities that may exploit the function of protease inhibitors.

Rha et al (1997) showed sequential production and activation of MMP-9 with breast cancer progression. In both DCIS and $T_1$ stage breast tumours they reported only 92 kDa (proMMP-9) gelatinolytic activity as compared to non-cancerous tissue but they found no difference in the 82 kDa (active form) activity among them. In the present study we cannot distinguish between these two events. The antibodies we used recognize the latent form of the enzyme. It is likely that MMP-9 present in tumours from node-negative patients is initially enzymatically inactive and as tumours become more aggressive the balance shifts towards the active form, thus the latent MMP-9 staining disappears. Present results are in agreement with Garbett et al (1999) who found the latent form of MMP-9 in both tumour and background breast tissues; however, they found only the active form of this enzymes in tumour tissue of paired samples. Furthermore, it is important to consider that MMP-9 may be under regulation by steroid hormones during mammary tumour progression, as the breast is a hormone-responsive tissue. The precise signaling pathways mediating MMP regulation by hormones are not well understood, but have been shown to involve direct interaction with AP-1 transcription factors in some cases (Jonat et al, 1990).

If expression of MMP-9 constitutes a host response to tumour invasion, this may alter hormonal status by triggering series of yet unknown pathways. Also, alteration to this protease may alter the balance between host proteases and protease inhibitors produced by the tumour cells enabling inhibition of matrix degradation and tumour invasion. The possible reason for the apparently paradoxical association of MMP-9 positivity with better outcome may be that in more aggressive tumors the MMP-9 may be in an active state and therefore have a shorter half-life. There may also be a greater amount of enzyme that is secreted (Hanemaaijer et al, 1999; Zucker et al, 1999) and not cell-associated and thus probably transparent to immunohistochemistry. Although for assessment of the role of MMPs in tumour progression, both enzyme and inhibitor levels must be analysed. However, activation of the latent precursor is also a key step in determining enzyme function in vivo. For activation of proMMP-9, the serine proteases are thought to be important and MT-MMP has been shown to be a potent activator of MMP-2. Jones et al (1999) examining immunohistochemically MMP-9, MMP-2 their inhibitors and the activator MT1-MMP reported a potentially important role for MT1-MMP which may be a pivotal factor for controlling MMP-2 activity but showed no relationship for TIMP-1 to tumour grade or lymph node status. The possible involvement of TIMPs/MT-MMPs is currently under investigation in our lab. Current therapeutic strategies using synthetic inhibitors of MMPs have demonstrated clinical potential of regulating protease activity and may be new targets for therapeutic exploitation.

### REFERENCES

Ardavanis A, Scorilas A, Amanatidou A, Gerakini F, Missitzis I, Garoufali A, Pissakas G, Pateras C, Apostolikas N, Rigatos G and Yiotis I (1997) Cathepsin-D concentration in tumour cytosols improves the accuracy of prognostic evaluation of primary breast cancer. *Anticancer Res* **17**: 1405–1410

Bajou K, Noel A, Gerard RD, Masson V, Brunner N, Holst-Hansen Skobe M, Fusenig NE, Carmeliet P, Collen D and Foidart JM (1998) Absence of host plasminogen activator inhibitor 1 prevents cancer invasion and vascularization. *Nat Med* **8**: 923–928

Benaud CH, Dickson RB and Thompson EW (1998) Roles of the matrix metalloproteinases in mammary gland development and cancer. *Br Cancer Res Treat* **50**: 97–116

Bianchi E, Cohen RL, Dai A, Thor AT, Shuman MA and Smith HS (1995) Immunohistochemical localization of the plasminogen activator inhibitor-1 in breast cancer. *Int J Cancer* **60**: 597–603

 *© 2001 Cancer Research Campaign*

Blavier L and DeClerck YA (1997) Tissue inhibitor of metalloproteinases-2 is expressed in the interstitial matrix in adult mouse organs and during embryonic development. *Molecular Biology of the Cell* **8**: 1513–1527

Bruner KL, Rodgers WH, Gold LI, Korc M, Hargrove JT, Matrisian LM and Osteen KG (1995) Transforming growth factor-b mediates the progesterone suppression of an epithelial metalloproteinase by adjacent stroma in the human endometrium *Proc Natl Acad Sci USA* **92**: 7362–7366

Chambers AF and Matrisian LM (1997) Changing views of the role of matrix metalloproteinases in metastasis. *J Natl Cancer Inst* **89**: 1260–1270

Daidone MG, Silverstrini R, D'Errico A, Di Fronza G, Benini E, Mancini A, Garbisa S Liotta L and Grigioni W (1991) Laminin receptors, collagenase IV and prognosis in node negative breast cancers. *Int J Cancer* **48**: 529–532

Dano K, Behrendt N, Brunner N, Ellis V, Plong M and Pyke C (1994) The urokinase receptor: protein structure and the role in plasminogen activation and cancer invasion. *Fibrinolysis* **8**: 189–203

Doussal VL, Tubiana-Hulin M, Friedman S, Hacerne K, Spyratos F and Brunet M (1989) Prognostic value of histologic grade nuclear components of Scarff-Bloom-Richardson (SBR): an improved score modification based on a multivariate analysis of 1262 invasive ductal breast carcinomas. *Cancer* **64**: 1914–1922

Duffy MJ, Blaser J, Duggan C, McDermott E, O'Higgins N, Fennelly JJ and Tschesche H (1995) Assay of matrix metalloproteinases types 8 and 9 by ELISA in human breast cancer. *Br J Cancer* **71**: 1025–1028

Elashry-Stowers D, Zava D, Speers W and Edwards D (1988) Immunochytochemical localization of progesterone receptors in breast cancer with antihuman receptor monoclonal antibodies. *Cancer Res* **48**: 6462–6474

Friedman R, Toth M, Pena D and Mobashery (1995) Activation of progelatinase B (MMP-9) by gelatinase A (MMP-2). *Cancer Res* **55**: 2548–2555

Garbett EA, Reed MWR and Brown NJ (1999) Proteolysis in human breast and colorectal cancer. *Br J Cancer* **81**: 287–293.

Hanemaaijer R, Sier C, Visser H, Scholte L, van Lent N, Toet K, Hoekman K and Verheijen J (1999) MMP-9 activity in urine from patients with various tumors, as measured by a novel MMP activity assay modified urokinase as a substrate. *Ann NY Acad Sci* **878**: 141–149

Heppner K, Matrisian L, Jensen R and Rodgers W (1996) Expression of most matrix metalloproteinase family members in breast cancer represents a tumor-induced host response. *Am J Pathol* **149**: 273–282

Hewitt RE and Dano K (1996) Stromal cell expression of components of matrix degrading protease systems in human cancer. *Enzyme Protein* **49**: 163–173

Jonat C, Rahmsdort HJ, Park K, Cato ACB, Gebel S, Ponta H and Herrlich P (1990) Anti-tumor promotion and anti-inflammation: down-modulation of AP-I (Fos/Jun) activity by glycocorticoid hormone. *Cell* **62**: 1189–1204

Jones J, Glynn P and Walker R (1999) Expression of MMP-2 and MMP-9, their inhibitors and the activator MT1-MMP in primary breast carcinomas. *J Pathol* **189**: 161–168

Kaplan EL and Meier P (1958) Nonparametric estimation from incomplete observations. *J Am Stat Assoc* **53**: 457–481

Karameris A, Panagou P, Tsilalis T and Bouros D (1997) Association of expression of metalloproteinases and their inhibitors with the metastatic potential of squamous-cell lung carcinomas. A molecular and immunohistochemical study. *Am J Respir Crit Care Med* **156**: 1930–1936

Kute TE, Hushe MS and Phyme AC (1980) Improvements in steroid receptor assays including rapid computer analysis of data. *Anal Biochem* **103**: 272–279

Liotta LA and Stetler-Steverson G (1991) Tumor invasion and metastasis: An imbalance of positive and negative regulation. *Cancer Res* **51**: 5054–5059

Magklara A, Scorilas A, Stephan C, Kristiansen G, Hauptmann S, Jung K and Diamandis EP (2000) Down-regulation of prostate specific antigen (PSA) and human glandular kallikrein 2 (hK2) in malignant vs non-malignant prostatic tissue. *Urology* **56**: 527–532

Mira E, Manes S, Lacalle RA, Marquer G and Mortinez C (1999) Insulin-like growth factor-1 triggered cell migration and invasion are mediated by matrix metalloproteinase-9. *Endocrinology* **140**: 1657–1664

Nelson A, Fingleton B, Rothenberg M and Matrisian L (2000) Matrix metalloproteinase: Biologic activity and clinical implications. *J Clin Oncol* **18**: 1135–1149

Nielsen BS, Timshel S, Kjeldsen L, Sehested, Pyke C, Borregaard N and Dano K (1996a) 92 kDa type IV collagenase (MMP-9) is expressed in neutrophils and macrophages but not in malignant epithelial cells in human colon cancer. *Int J Cancer* **65**: 57–62

Nielsen BS, Sehesed M, Timshel S, Pyke C and Dano K (1996b) Messenger RNA for urokinase plasminogen activator (uPA) is expressed in myofibroblasts adjacent to cancer cells in human breast cancer. *Lab Investigations* **74**: 168–177

Nielsen BS, Sehested M, Kjeldsen L, Borregaard N, Rygaard J and Danon K (1997) Expression of matrix metalloprotease-9 in vascular pericytes in human breast cancer. *Lab Investigation* **77**: 345–355

Pacheco MM, Mourao M, Edson BM, Nishimoto IN and Brentani MM (1998) Expression of gelatinases A and B, stromelysin-3 and matrilysin genes in breast carcinomas: clinico-pathological correlations. *Clin Exp Metastasis* **16**: 577–585

Ree AH, Bjørnland K, Brünner N, Johansen HT, Pedersen KB, Aasen AO and Fodstad O (1998) Regulation of tissue-degrading factors and in vitro invasiveness in progression of breast cancer cells. *Clin Exp Metastasis* **16**: 205–215

Remacle AG, Noël A, Duggan C, McDermott E, O'Higgins N, Foidart JM and Duffy MJ (1998) Assay of matrix metalloproteinases types 1,2,3 and 9 in breast cancer *Br J Cancer* **77**: 926–931

Rha SY, Kim JH, Roh JK, Lee KS, Min JS, Kim BS and Chung HC (1997) Sequential production and activation of matrix-metalloproteinase-9 (MMP-9) with breast cancer progression. *Br Cancer Res Treatment* **43**: 175–181

Rha SY, Yang WI, Kim JH, Roh JK, Min JS, Lee KS, Kim BS and Chung HC (1998) Different expression patterns of MMP-2 and MMP-9 in breast cancer. *Oncol Rep* **5**: 875–879

Salamonsen LA, Butt AR, Hammond R, Garcia S and Zhang J (1997) Production of endometrial matrix metalloproteinases, but not their tissue inhibitors are modulated by progesterone withdrawal in an in vitro model for menstruation. *J Clin Endocrinol Metab* **78**: 1409–1415

Scorilas A, Yotis J, Gouriotis D, Keramopoulos A, Ampela K, Trangas T and Talieri M (1993) Cathepsin-D and c-erbB-2 have an additive prognostic value for breast cancer patients. *Anticancer Res* **13**: 1895–1900

Scorilas A, Yotis J, Stravolemos K, Gouriotis D, Keramopoulos A, Ampela K, Talieri M and Trangas T (1995) c-erB-2 overexpression may be used as an independent prognostic factor for breast cancer patients. *Anticancer Res* **15**: 1543–1548

Scorilas A, Yotis J, Pateras Ch, Trangas Th and Talieri M (1999a) Predictive value of c-erbB-2 and Cathepsin-D for Greek breast cancer patients using univariate and multivariate analysis. *Clin Cancer Res* **5**: 815–821

Scorilas A, Trangas Th, Yotis J, Pateras Ch and Talieri M (1999b) Determination of c-myc amplifications and overexpression in breast cancer patients. Evaluation of its prognostic value against c-erbB-2, cathepsin-D and clinicopathologic characteristics using univariate and multivariate analysis. *Br J Cancer* **81**: 1385–1391

Scorilas A, Diamandis E, Levesque M, Papanastasiou-Diamandis a, Khoszavi M, Giai M, Ponzone R, Roagua R, Sismondi P and López-Otin C (1999c) Immuno-enzymatically determined pepsinogen C concentration in breast tumor cytosols: An independent favorable prognostic factor in node-positive patients. *Clin Cancer Res* **5**: 1778–1785

Scorilas A, Talieri M, Ardavanis A, Courtis N, Yotis J, Dimitriadis E, Tsiapalis C and Trangas T (2000a) Polyadenylate polymerase enzymatic activity in mammary tumor cytosols: A new independent prognostic marker in primary breast cancer. *Cancer Res* **60**: 5427–5433

Scorilas A, Black M, Talieri M and Diamandis E (2000b) Genomic organization, physical mapping and expression analysis of the human protein arginine methyltransferase 1 gene. *Biochem Biophys Res Commun* **278**: 349–359

Spiessl B, Beahrs OH, Hermanek P, Hutter RVP, Scheibe O, Sobin LH and Wagner G (1989) *Illustrated guide to the TNM/p TNM classification of malignant tumours. TNM Atlas*, 3rd edn

Strongin AY, Collier I, Bannikov G, Marmer BL, Grant GA and Goldberg GI (1995) Mechanisms of cell surface activation of 72-kDa type IV collagenase *J Biol Chem* **270**: 1–8

Sugiura Y, Shimada H, Seeger R, Lang W and Declerck YA (1998) Matrix metalloproteinases-2 and -9 are expressed in human neuroblastoma: Contribution of stromal cells to their production and correlation with metastasis. *Cancer Res* **58**: 2209–2216

Takeha S, Fujiyama Y, Bamda T, Sorsa T, Nagura H and Ohtani H (1997) Stromal expression of MMP-9 and urokinase receptor is inversely associated with liver metastasis and with infiltrating growth in human colorectal cancer: A novel approach from immune/inflammatory aspect. *Jpn J Cancer* **88**: 72–81

Thorpe SM, Christensen IJ, Rasmussen BB and Rose C (1993) Short recurrence-free survival associated with high oestrogen receptor levels in the natural history of postmenopausal primary breast cancer. *Eur J Cancer* **29A**: 971–977

Tryggvason K, Hoyhtya M and Pyke C (1993) Type IV collagenase in invasive tumors. *Br Cancer Res Treat* **24**: 209–218

Visscher DW, Hoyhtya M, Ottosen SK, Liang C-M, Sarkar FH, Crissman JD and Fridman R (1994) Enhanced expression of tissue inhibitor of metalloproteinase-2 (TIMP-2) in the stroma of breast carcinomas correlates with tumor recurrence. *Int J Cancer* **59**: 339–344

© 2001 Cancer Research Campaign

Vizoso F, Sanchez LM, Diez-Itza I, Merino AM and Lopez-Otin C (1995) Pepsinogen C is a new prognostic marker in primary breast cancer. *J Clin Oncol* **13**: 54–61

Vu TH and Werb Z (1998) Gelatinase B Structure, regulation and function. In: *Matrix metalloproteinases*, Parks WC and Mecham RP (eds) pp 115–148. Academic Press: San Diego, CA

Yan S, Sameni M and Sloane BF (1998) Cathepsin B and human tumor progression *Biol Chem* **379**: 113–123

Yousef GM, Scorilas A and Diamandis EP (2000) Genomic organization, mapping, tissue expression and hormonal regulation of trypsin-like serine protease (TLSP PRSS20), a new member of the human kallikrein gene family. *Genomics* **63**: 88–96

Yousef G, Scorilas A, Jung K, Aschworth L and Diamandis EP (2001) The new human kallikrein gene KLK15 is up-regulated in prostate cancer. *J Biol Chem* **276**: 53–61

Yu H, Levesque MA, Clark GM and Diamandis EP (1998) Prognostic value of prostate-specific antigen for women with breast cancer: a large United States cohort study. *Clin Cancer Res* **4**: 1489–1497

Yu Q and Stamenkovic (1999) Localization of matrix metalloproteinase 9 to the cell surface provides a mechanism for CD44-mediated tumor invation. *Genes Dev* **13**: 35–48

Zucker S, Hymowitz M, Conner C, Zarrabi H, Hurewitz A, Matrisian L, Boyd D, Nicolson G and Montana S (1999) Measurement of matrix metalloproteinases and tissue inhibitors of metalloproteinases in blood and tissues. *Ann NY Acad Sci* **878**: 212–227

*© 2001 Cancer Research Campaign*
